# Tumor mutational burden as a determinant of metastatic dissemination patterns

**DOI:** 10.1002/1878-0261.70200

**Published:** 2026-01-27

**Authors:** Eduardo Candeal, Andrea Moreno‐Manuel, Miguel Salvadó‐Pertierra, Cristina Santos‐Vivas, Rebeca Sanz‐Pamplona

**Affiliations:** ^1^ Cancer Heterogeneity and Immunomics (CHI) Clinical University Hospital Lozano Blesa, Aragon Health Research Institute (IISA) Zaragoza Spain; ^2^ Medical Oncology Department Catalan Institute of Oncology (ICO) – ONCOBELL Barcelona Spain; ^3^ Consortium for Biomedical Research in Oncology (CIBERONC) Madrid Spain; ^4^ Aragonese Foundation for Research and Development (ARAID) Zaragoza Spain; ^5^ Consortium for Biomedical Research in Epidemiology and Public Health (CIBERESP) Madrid Spain

**Keywords:** immunotherapy, metastasis, mutations, organotropism, prognostic biomarker, TMB

## Abstract

According to the seed and soil hypothesis, the organ specificity of metastasis is not a random process and depends on multiple tumor‐intrinsic and microenvironmental factors. In this study, we characterized the mutational landscape of a large cohort of human metastatic samples to investigate whether mutational trends determine metastatic dissemination. Genomic data from nine cancer types (bladder, breast, colorectal, endometrial, melanoma, non‐small cell lung cancer, ovarian, pancreatic, and prostate) including 19827 patients were obtained from a pan‐cancer study. When restricting the analysis to driver mutations, no robust, recurrent mutational patterns associated with metastatic locations were identified across cancer types. However, when cancer types were analyzed separately, mutational trends associated with specific metastatic locations emerged. Considering the total tumor mutational burden (TMB), central nervous system (CNS)/brain and lung metastases harbored a higher TMB than other metastatic locations. Since higher TMB in CNS/brain metastases was also associated with improved prognosis, these findings may be pivotal in refining immunotherapy strategies. Indeed, this observation was confirmed in an independent dataset including patients treated with immunotherapy. In conclusion, our findings suggest that TMB may have greater influence on metastatic organotropism than driver mutational background.

## Graphical abstract

This study performed a comprehensive analysis of genomic data to elucidate whether metastasis in certain organs share genetic characteristics regardless of cancer type. No robust mutational patterns were identified across different metastatic locations and cancer types. By contrast, we observed that tumor mutational burden varied across metastatic sites and its prognostic value differed depending on the tissue sample analyzed.

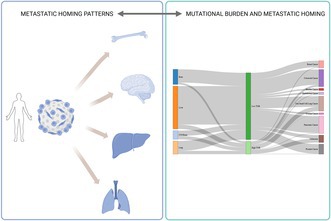

**Abbreviations**
APC: adenomatous polyposis coli
ARID1A: AT‐rich interaction domain 1A
ATM: ataxia telangiectasia mutated
BRAF: B‐Raf proto‐oncogene, serine/threonine kinase
BRCA2: breast cancer type 2 susceptibility protein
CBFB: core‐binding factor subunit beta
CDH1: cadherin 1 (E‐Cadherin)
CNS: central nervous system
EGFR: epidermal growth factor receptor
EP300: E1A binding protein P300
EPHA5: ephrin type‐A receptor 5
FAT1: FAT atypical cadherin 1
FBXW7: F‐Box and WD repeat domain containing 7
FOXA1: Forkhead box A1
GRIN2A: Glutamate Ionotropic receptor NMDA type subunit 2A
KMT2C: Lysine methyltransferase 2C (MLL3)
MAP2K2: mitogen‐activated protein kinase kinase 2 (MEK2)
MAP2K4: mitogen‐activated protein kinase kinase 4 (MEK4)
MED12: Mediator complex subunit 12
Mut/Mb: mutations per megabase
NF1: Neurofibromin 1
NRAS: Neuroblastoma RAS viral oncogene homolog
NSCLC: non‐small cell lung cancer
OS: overall survival
PC: principal component
PCA: principal component analysis
PIK3CA: Phosphatidylinositol‐4,5‐bisphosphate 3‐kinase catalytic subunit alpha
PTPRD: protein tyrosine phosphatase receptor type D
PTPRT: protein tyrosine phosphatase receptor type T
RB1: Retinoblastoma 1
SETD2: SET domain containing 2 (histone methyltransferase)
TMB: tumor mutational burden
ZFHX3: Zinc finger homeobox 3

## 1 Introduction

Metastatic disease remains the leading cause of cancer‐related mortality, yet its underlying molecular mechanisms are still not fully understood [1]. Although the general steps of metastasis are similar across most cancer types, distinct sets of regulators may be required for tumor cell infiltration and colonization in specific tissues [2]. The ‘seed and soil’ hypothesis proposes that metastatic growth in distant organs results from favorable interactions between disseminated tumor cells from the primary site (the seed) and the microenvironment of the target organ (the soil) [3]. According to this hypothesis, metastatic colonization is a non‐random process, and different cancer types exhibit distinct patterns of organ‐specific metastasis, known as organotropism [4].

In his seminal work, Massagué and colleagues described the genetic determinants of metastatic tropism in breast cancer. They identified a set of genes that predict the metastatic potential of breast cancer to various organs. Some of these genes confer growth advantages in both the primary tumor and the microenvironment of the target organ, while others promote aggressive growth specifically in metastatic sites [5, 6, 7]. Furthermore, a direct link between genetic alterations in cancer cells and remodeling of the tumor immune microenvironment has been extensively described. In metastatic sites, cancer cells encounter a hostile immune microenvironment, and growing evidence highlights the role of the metastatic niche in facilitating immune evasion [8, 9, 10].

In a previous study, our group reported that lung metastases exhibited higher immunogenicity compared to metastases in other organs, regardless of the primary cancer type [11]. This observation, based on transcriptomic data, suggested a potential organ‐specific immune contexture associated with increased immune activity. Building on these findings, we sought to explore whether this pattern could be confirmed at the genomic level. We hypothesized that the mutational background—including both recurrently mutated genes and tumor mutational burden (TMB)—may influence organ specificity in metastasis by modulating specific pathways and immune cells interactions within the metastatic niche. To investigate this, we analyzed the mutational landscape of metastatic tumor samples and examined their correlation with the anatomical distribution of metastases. Our aim was to determine whether specific genomic features are linked to the propensity of tumors to colonize specific organs, thus offering novel insights into the molecular determinants of metastatic tropism.

## 2 Materials and methods

### 2.1 Samples

Clinical and genomic data from 25 775 samples (15 632 metastatic and 10 143 primary tumor samples) from cancer patients were downloaded from cBioPortal [12]. Only those cancer types with at least 500 cases were selected for the study. Nine cancer types were included: bladder cancer, breast cancer, colorectal cancer (CRC), endometrial cancer, melanoma, non‐small cell lung cancer (NSCLC), ovarian cancer, pancreatic cancer, and prostate cancer. For inclusion in our analysis, metastatic locations within each cancer type had to contain at least 20 samples. The selected metastatic sites included the following: adrenal gland, bone, central nervous system (CNS)/brain, intra‐abdominal, kidney, liver, lung, lymph nodes (both locoregional and distant nodes), mediastinum, ovary, pleura, skin, and others. The ‘others’ category encompassed metastases in the biliary tract, bladder/urinary tract, bowel, breast, female genital tract (excluding ovary), head and neck, and male genital tract.

The final cohort consisted of 19 827 samples (Table S1), including 12030 primary tumor samples (8736 from metastatic and 3294 from non‐metastatic patients) and 7797 metastatic samples (hereafter referred to as the Nguyen *et al*. dataset). For principal component analysis (PCA), multivariate modeling, and TMB analyses, we only focused on the most common distant metastases associated with hematogenous dissemination (*n* = 3386): bone (*n* = 595), CNS/brain (*n* = 353), liver (*n* = 1874), and lung (*n* = 564). Mutational data were retrieved for all included patients, who had been sequenced using MSK‐IMPACT panels covering 341, 410, or 468 genes.

To validate the TMB results, we obtained an independent dataset comprising 1661 patients treated with immunotherapy (hereafter referred to as the Samstein *et al*. dataset), also from cBioPortal [13]. We selected a subset of 590 metastatic patients, including primary tumor samples (*n* = 338), and bone (*n* = 32), CNS/brain (*n* = 40), liver (*n* = 87), and lung (*n* = 93) metastatic samples. All selected samples had been analyzed using the MSK‐IMPACT410 panel, which was the most frequent in this dataset. Baseline characteristics of these patients were detailed in Table S2.

### 2.2 Principal component analysis (PCA) and hierarchical clustering using recurrently mutated genes

The frequency of recurrently mutated genes across sample sites was calculated by dividing by the total number of samples in the respective group. For each of the most frequent distant hematogenous metastatic locations—bone, CNS/brain, liver, and lung—the 20 most frequently mutated genes within each cancer type were identified and subsequently combined. This resulted in varying numbers of genes to analyze: 63 for bone, 61 for CNS/brain, 101 for liver, and 93 for lung metastases, a total of 142 genes. These genes were then used to perform PCA and hierarchical clustering. Next, a stratified analysis was carried out using the top 20 genes with the highest mutational frequency across all samples within each cancer type (Fig. S1), resulting in 93 genes. Dendrograms were generated using hierarchical clustering based on Euclidean distance.

### 2.3 Multivariate analysis

To evaluate whether metastatic site could be predicted based on mutational profiles, we conducted multivariate modeling using the Nguyen *et al*. dataset. As explained before, only patients with the most frequent hematogenous metastases were included. The resulting cohort (*n* = 3386) was randomly split into a training set (*n* = 2372 patients, 70%) and a test set (*n* = 1014 patients, 30%). Genes mutated in at least 20 patients were retained for inclusion in the input matrix. To ensure robustness, fivefold cross‐validation and three independent iterations of the entire modeling process were performed to minimize variability. Three different modeling approaches were applied to ensure reliability of the results. The first model was developed using XGBoost and the xgbTree method from the *caret* package, an ensemble approach based on decision tree algorithms. This model was designed to capture non‐linear relationships and complex interactions within the mutational data. The second model employed an elastic net approach, created using the *glmnet* package. This method is particularly suited for high‐dimensional datasets, as it performs variable selection through a regularization technique that combines both Lasso (L1) and Ridge (L2) penalties. To fine‐tune the model, the hyperparameters α and λ were optimized using a tune Length of 10, which allowed exploration of ten distinct parameter combinations. The third model was constructed using a Random Forest classifier from the *caret* package. This model was built with default parameters, including 500 decision trees, with 18 predictors randomly selected at each split.

### 2.4 Tumor mutational burden analysis

TMB was defined as the number of mutations per megabase (Mut/Mb). TMB was evaluated using the Nguyen *et al*. dataset, focusing on the subset of metastatic patients (*n* = 8438). Since gene panel size influences TMB estimation, to ensure consistency only samples sequenced with the MSK‐IMPACT468 gene panel were analyzed. These samples comprised primary tumors (*n* = 6373), as well as bone (*n* = 391), CNS/brain (*n* = 225), liver (*n* = 1109), and lung (*n* = 340) metastases. Boxplots of log_10_‐transformed TMB values were generated to facilitate visualization. Median TMB was used to generate heatmaps to highlight the metastatic sites with higher TMB variation across cancer types. To depict the distribution of high and low TMB across cancer types and metastatic sites, a Sankey diagram was constructed. When dichotomization was required, a threshold of 10 mutations per megabase (Mut/Mb) was applied, as it represents the tumor‐agnostic cut‐off established as a predictive biomarker for immunotherapy responsiveness in solid tumors [14].

### 2.5 Statistical analysis

Data were downloaded and analyzed using RStudio v2022.02.1 (R version 4.1.3). The distribution and homoscedasticity of variables were assessed using Kolmogorov–Smirnov and Levene's tests, respectively. Based on these results, non‐parametric tests such as Kruskal–Wallis and Dunn tests were used. Overall survival analysis was conducted using univariate Cox proportional hazards regression, and survival curves were represented using Kaplan–Meier plots, with significance assessed by log‐rank test. To identify the most distinctive mutated genes across sample sites, Grubbs' test for outliers was performed. Statistical significance was considered when the *P*‐value was below 0.05.

## 3 Results

### 3.1 Patterns of metastatic dissemination across cancer types

**Fig. 1:**
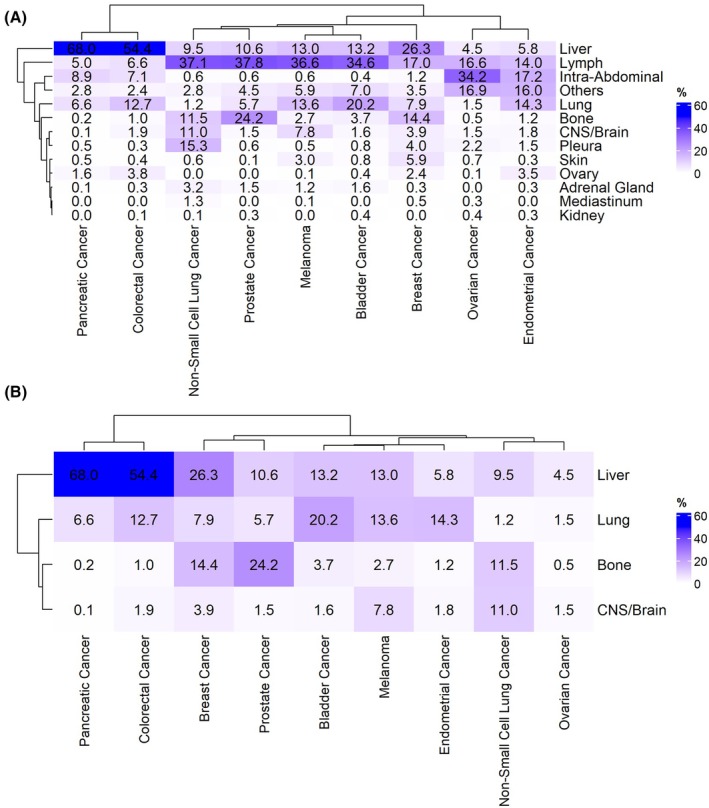
Metastatic homing footprint. (A) Heatmap representing frequency of metastatic sites across cancer types. Category others includes biliary/urinary tract, bowel, bladder, breast, female genital (excluding ovary), head and neck, and male genital metastases. (B) Heatmap showing the frequency of the four more frequent distant metastases (bone, CNS/brain, liver, and lung) across cancer types. Sample size was 6883 (A) and 3454 (B) patients. Color scale was determined by cell, based on frequency of the metastasis (blue higher frequency, white lower frequency). CNS, central nervous system.

Frequencies of metastatic colonization across the nine cancer types were shown in Fig. 1A, Table S3, and Fig. S2. The distribution aligned with previous reports [15, 16, 17]. For instance, dendrograms grouped CRC and pancreatic cancer together, both displaying liver tropism. Prostate cancer mainly metastasized to bone. Bladder, melanoma, prostate, and lung cancer clustered due to their high incidence of lymph node metastases. Gynecological tumors—including endometrial and ovarian—also exhibited similar dissemination patterns, although endometrial cancer showed a higher frequency of lung metastases. Breast cancer displayed a distinctive distant homing signature, predominantly metastasizing to liver, lungs, and bone.

Focusing on the four most frequent distant hematogenous metastatic locations—bone, CNS/brain, liver, and lung—interesting patterns emerged (Fig. 1B). Liver metastases separated from the other metastases in the dendrogram, primarily originating from CRC and pancreatic tumors, followed by breast cancer. In contrast, lung metastases were mainly derived from bladder, endometrial, melanoma, and CRC. Bone and CNS/brain metastases presented different patterns but clustered together. Prostate cancer was the main source of bone metastases, followed by breast and lung cancer. Conversely, CNS/brain metastases predominantly originated from NSCLC, followed by melanoma and breast cancer.

### 3.2 Mutational patterns in the most frequent metastatic locations

**Fig. 2:**
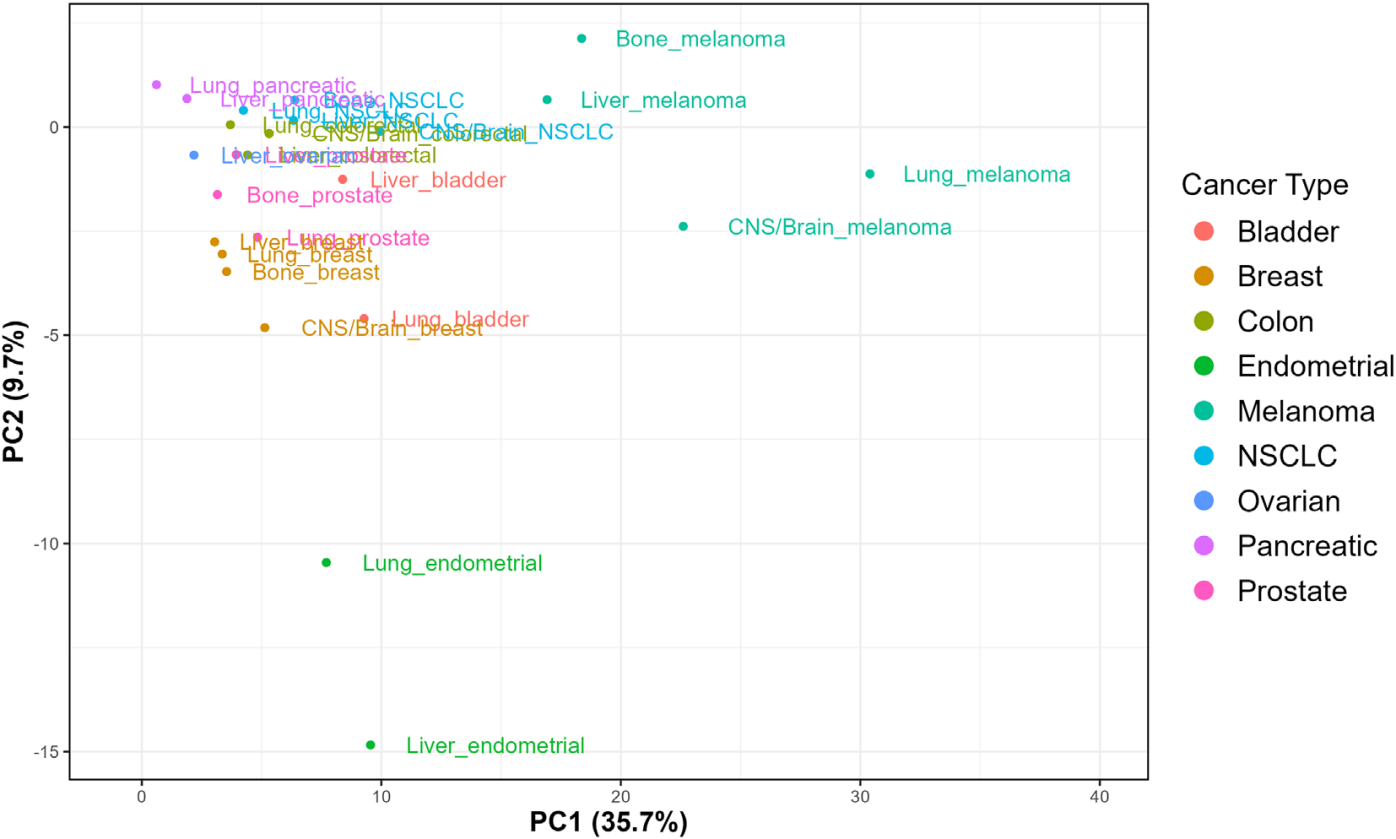
Principal Component Analysis of bone, CNS/brain, liver, and lung metastases across cancer types. PCA was generated including 3378 patients and the top 20 genes. NSCLC, non‐small cell lung cancer; PC, principal component; PCA, principal component analysis.

To assess whether recurrent mutational profiles were shared by metastatic samples, we first performed PCA based on the frequency of recurrently mutated genes. As illustrated in Fig. 2, metastases mainly clustered according to their cancer type of origin and not by metastatic location. This suggests that cancer type plays a greater role in metastases organotropism than mutation patterns. However, melanoma and endometrial tumors were particularly separated along Principal Component (PC) 1 and PC 2, respectively. CNS/brain metastases originating from breast cancer and NSCLC also diverged from the other metastatic samples, perhaps suggesting unique genomic characteristics.

**Fig. 3:**
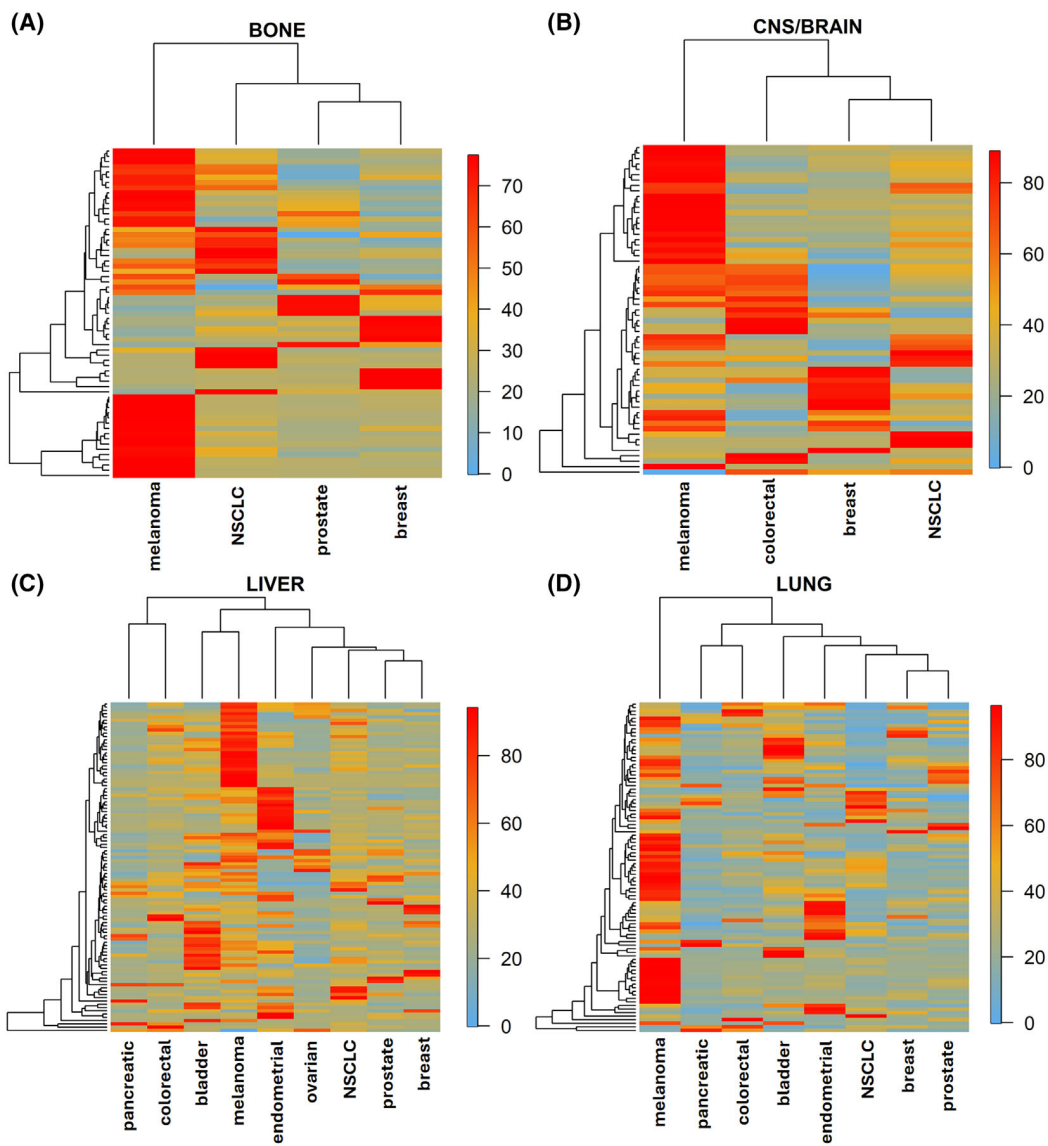
Mutational patterns across metastatic locations. Heatmaps based on mutational patterns of (A) bone, (B) CNS/brain, (C) liver, and (D) lung metastases across cancer types. Heatmap was generated including 3378 patients and the top 20 genes. Color scale was determined by row (blue lower frequency, red higher frequency). CNS, central nervous System; NSCLC, non‐small cell lung cancer.

Next, heatmaps and dendrograms were generated to cluster bone, CNS/brain, liver, and lung metastases based on the most frequently mutated genes (Fig. 3A–D). In agreement with the PCA results, lung, CNS/brain, and bone metastases derived from melanoma displayed distinctive mutational profiles. Interestingly, liver metastases from melanoma clustered together alongside those from bladder cancer. Nonetheless, no consistent clustering patterns were observed overall, suggesting that mutational profiles could be more strongly influenced by the primary cancer type than by site‐specific adaptation in distant organs.

To validate these observations, machine learning models were developed. Consistently, none of the three models tested—XGBoost, elastic net, or random forest—reached robust performance, with an average accuracy of 0.57 (Table S4). These results suggested that no enrichment of specific mutations was present in any metastatic site. Mutational profiles are insufficient for reliably classifying metastatic sites and are primarily governed by the characteristics of the originating tumor rather than by organ‐specific genomic adaptation.

### 3.3 Mutational patterns stratifying by cancer type

**Fig. 4:**
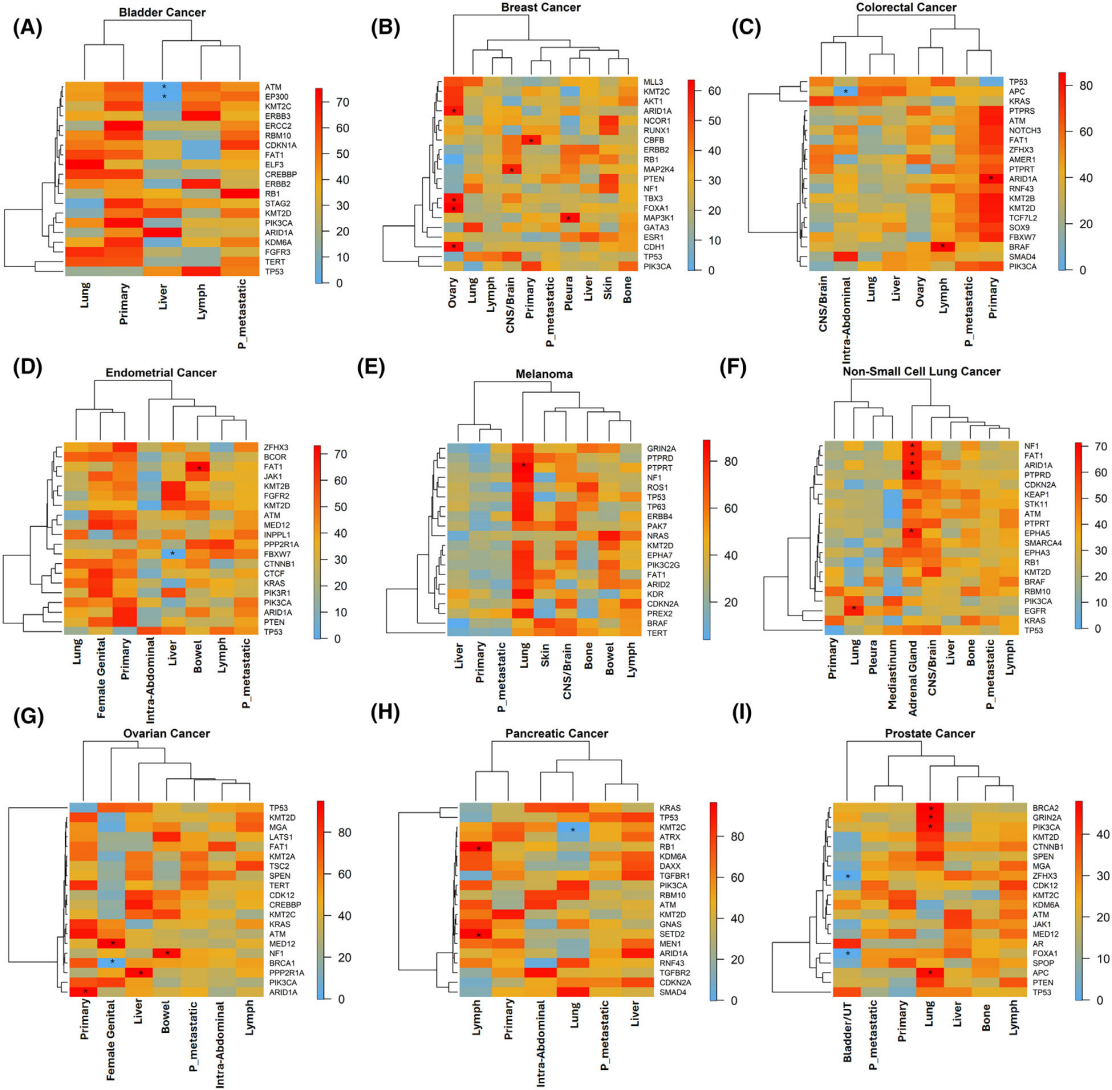
Gene mutation frequency across sample sites by cancer types. Heatmaps display the top 20 genes across cancer types (A–I), including both samples from primary tumors (Primary refers to primary nonmetastatic and P_metastatic to primary metastatic) and metastases. Sample size was 18 452 patients. *P*‐value was obtained using Grubb's statistical test. Color scale was determined by row (blue lower frequency, red higher frequency). CNS, central nervous system; NSCLC, non‐small cell lung cancer; P_metastatic, primary metastatic; UT, urinary Tract; *, *P* < 0.05.

Our results suggested that no mutations were consistently shared by metastases within the same organ. We then explored whether specific mutational patterns emerged across metastatic sites when stratifying by cancer type. Primary tumor samples were included in this analysis for comparison purposes and divided into primary tumors from metastatic patients (primary metastatic) and primary tumors from non‐metastatic patients (primary non‐metastatic). Figure 4A–I revealed distinct mutations associated with specific metastatic sites when each cancer type was analyzed individually. In NSCLC, *EGFR* mutations were overrepresented in lung metastases. Adrenal gland metastases were enriched for *NF1*, *FAT1*, *ARID1A*, *PTPRD*, and *EPHA5* mutations. Interestingly, primary non‐metastatic tumors clustered with pleura, mediastinum, and lung sites, whereas primary metastatic tumors clustered with distant metastatic locations. In breast cancer, CNS/brain metastases were enriched for *MAP2K2* mutations. *CBFB* mutations were predominantly found in primary non‐metastatic tumors, suggesting a less aggressive phenotype. In CRC, primary non‐metastatic tumors generally exhibited an enrichment for recurrent mutations, although only *ARID1A* was statistically significant. This may indicate the better prognosis typically associated with hypermutated tumors. Lymph node metastases were enriched for *BRAF* mutations, while intra‐abdominal metastases showed an underrepresentation of *APC* mutations. In melanoma, an interesting trend was observed; both primary non‐metastatic and primary metastatic tumor samples clustered with liver metastases. Lung metastases displayed a broader set of mutated genes, although only *PTPRT* reached statistical significance. In pancreatic cancer, lymph node metastases were enriched for *RB1* and *SETD2* mutations, whereas lung metastases exhibited a marked absence of *KMT2C* mutations. In prostate cancer, *BRCA2*, *GRIN2A*, *PIK3CA*, and *APC* were frequently mutated in lung metastases. Conversely, *FOXA1* and *ZFHX3* mutations were less common in bladder metastases. In ovarian cancer, *ARID1A* mutations were overrepresented in primary non‐metastatic tumors, suggesting a potential protective role. The *MED12* gene was clearly associated with genital relapse. In endometrial cancer, two genes reached statistical significance: *FAT1* was frequently mutated in bowel metastases, while *FBXW7* mutations were underrepresented in liver metastases. Finally, in bladder cancer, liver metastases exhibited lower mutation frequencies in *ATM* and *EP300* compared to other metastatic locations. For more details, the frequencies of all these genes in each sample type are shown in Table S5.

### 3.4 Association between tumor mutational burden and metastatic dissemination patterns

**Fig. 5:**
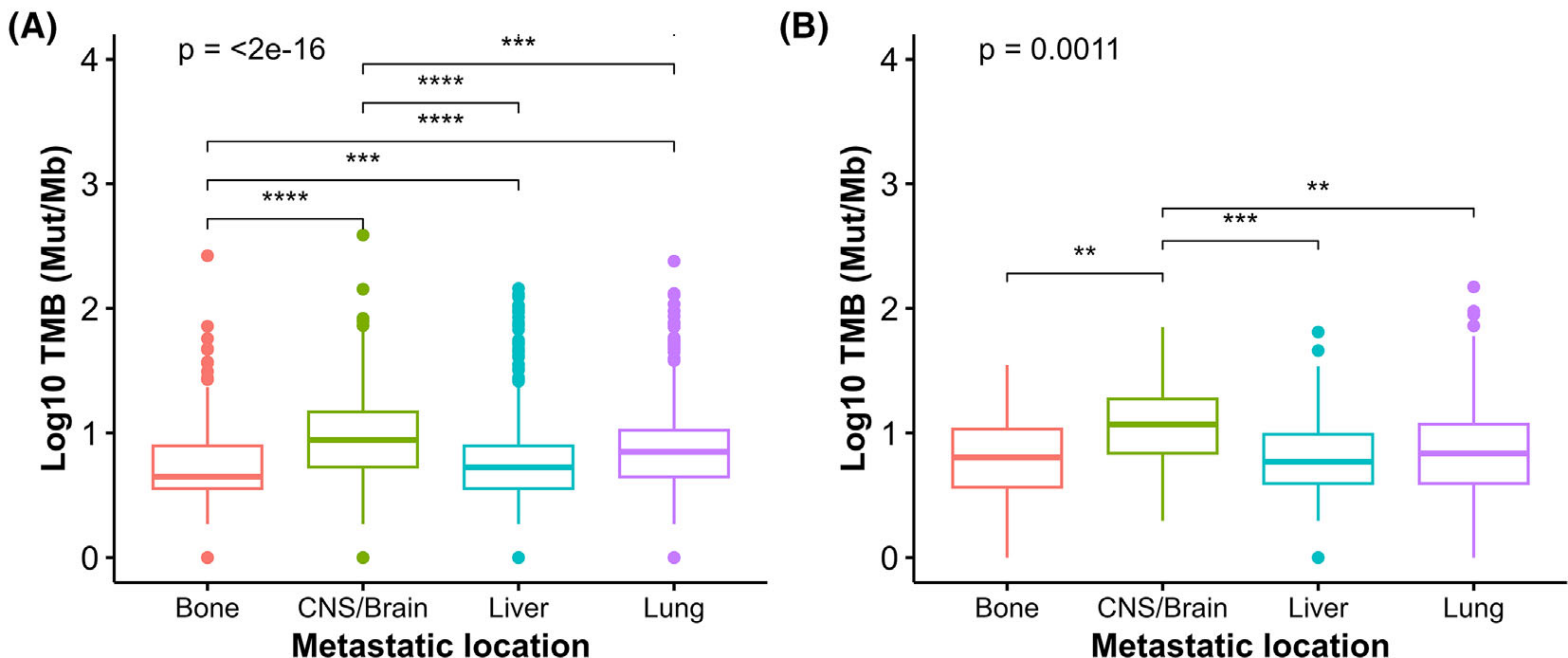
TMB patterns across metastatic locations. Boxplots of log_10_ TMB (Mut/Mb) according to metastatic location in the (A) Nguyen *et al*. dataset and (B) Samstein *et al*. dataset. Sample size was 2065 (A) and 252 (B) patients. *P*‐value was obtained using Kruskal–Wallis test. CNS, central nervous system; Mb, megabase; Mut, mutation; TMB, tumor mutational burden; **, *P* < 0.01; ***, *P* < 0.001; ****, *P* < 0.0001.

Following the identification of recurrently mutated genes, we investigated whether TMB as a measure of global mutational load might be associated with distinct patterns of metastatic dissemination. To address this, TMB was compared across the most frequent distant hematogenous metastatic sites: bone, CNS/brain, liver, and lung. We observed that metastases in CNS/brain and lung exhibited significantly higher TMB values compared to liver and bone metastases (Fig. 5A and Table S6). These findings were corroborated in the Samstein *et al*. dataset, in which CNS/brain metastasis had higher TMB than other metastatic locations. A non‐statistically significant trend was also observed in lung metastatic samples (Fig. 5B and Table S6).

One might hypothesize that the higher TMB in CNS/brain and lung metastatic samples may be influenced by the primary cancer type. Indeed, both melanoma and NSCLC tumors, known to exhibit elevated baseline TMB, were particularly enriched in CNS/brain and lung metastases. On the contrary, breast and pancreatic tumors with lower TMB were more prone to metastasize in the liver. This is consistent with the pattern of metastatic distribution seen in Nguyen *et al*. dataset (Fig. S2). To show primary tumor influence, boxplots stratifying by cancer type were depicted (Fig. S3 and Table S7).

**Fig. 6:**
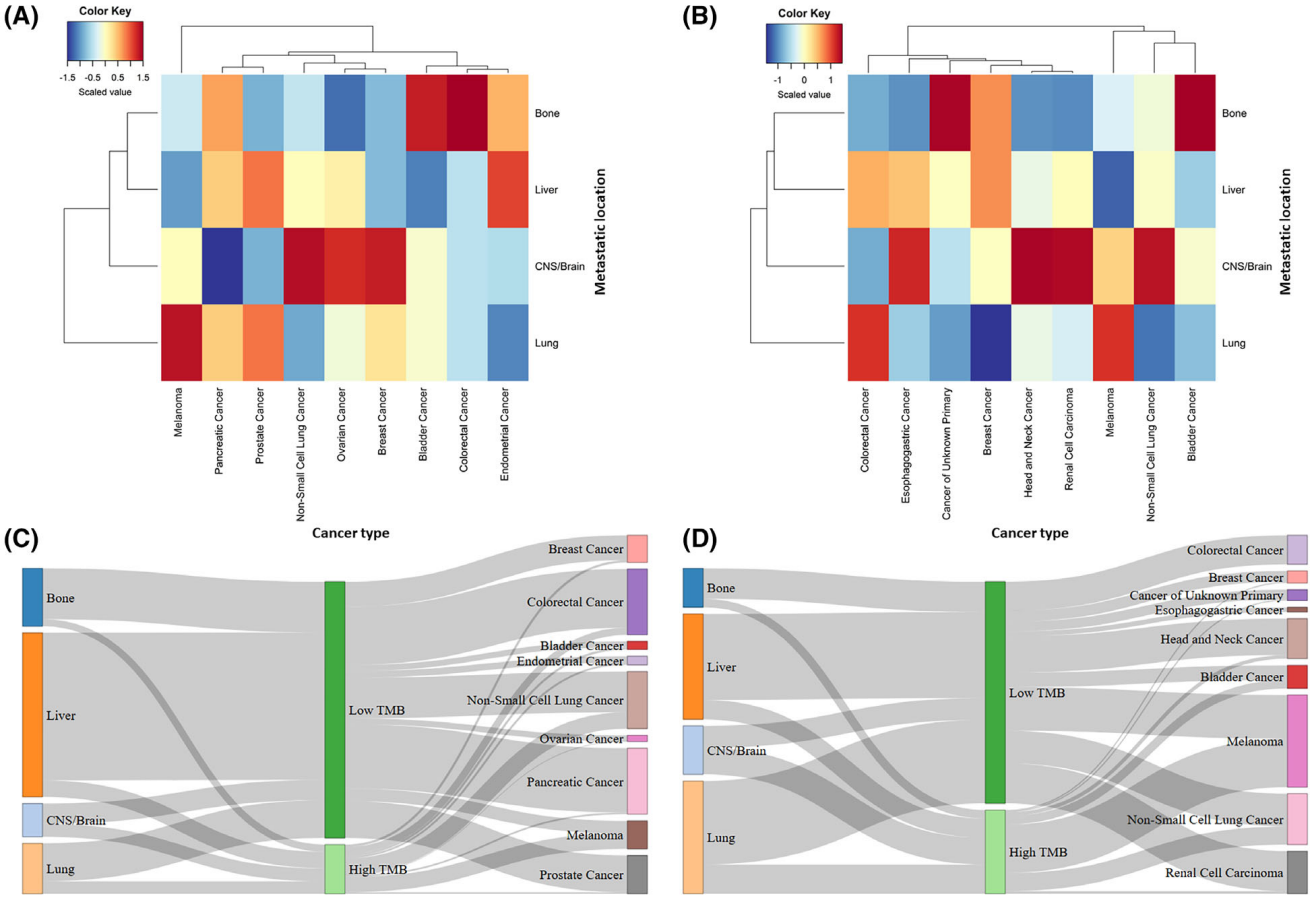
TMB patterns across metastatic locations and cancer types. Heatmaps showing TMB of metastatic samples across cancer types in the (A) Nguyen *et al*. and (B) Samstein *et al*. dataset. Median TMB (Mut/Mb) was used to generate the heatmap. Color scale was determined by column, to highlight the location with higher median TMB for each cancer type (blue lower TMB, red higher TMB). Sankey diagrams connecting TMB (dichotomized using 10 Mut/Mb as a cut‐off value) to metastatic sites and cancer types of metastatic samples in the (C) Nguyen *et al*. and (D) Samstein *et al*. dataset. Sample size was 2065 (A, C) and 252 (B, D) patients. CNS, central nervous system; Mb, megabase; Mut, mutation; TMB, tumor mutational burden.

To further explore these differences, we represented in a heatmap the median TMB for each metastatic site and cancer type. Notably, lung metastases were the more dissimilar branch in the dendrogram whereas bone and liver clustered together. CNS/brain metastases were the metastatic location with higher TMB in NSCLC, ovarian, and breast cancer (Fig. 6A). Most of these findings were validated in Samstein *et al*. including high TMB in lung metastases from melanoma. CNS/brain metastases were the ones with higher TMB in esophagogastric cancer, head and neck cancer, renal cell carcinoma, and NSCLC (Fig. 6B).

Finally, Sankey diagrams were depicted for illustrating the relationships between metastatic sites, cancer type, and TMB, in both patient cohorts (Fig. 6C,D). While most metastases displayed low TMB, site‐specific analysis revealed that approximately 50% of CNS/brain metastases had high TMB, followed by lung metastases. In contrast, liver and bone metastases were predominantly characterized by low TMB. Regarding cancer type, NSCLC, and melanoma, as expected, were the ones with higher TMB. To complement these observations, we compared the frequency of samples with high and low TMB across cancer types (Fig. S4). In the Nguyen *et al*. dataset, there was a statistically significant higher frequency of high TMB in CNS/brain metastases from melanoma, NSCLC, ovarian, and breast cancer. Also, in lung metastases from melanoma, ovarian, and prostate cancer patients. Consistently, in the Samstein *et al*. dataset, a higher frequency of samples with high TMB was observed in CNS/brain metastases from melanoma and NSCLC, as well as lung metastases from melanoma.

### 3.5 TMB and prognosis in metastatic samples

**Fig. 7:**
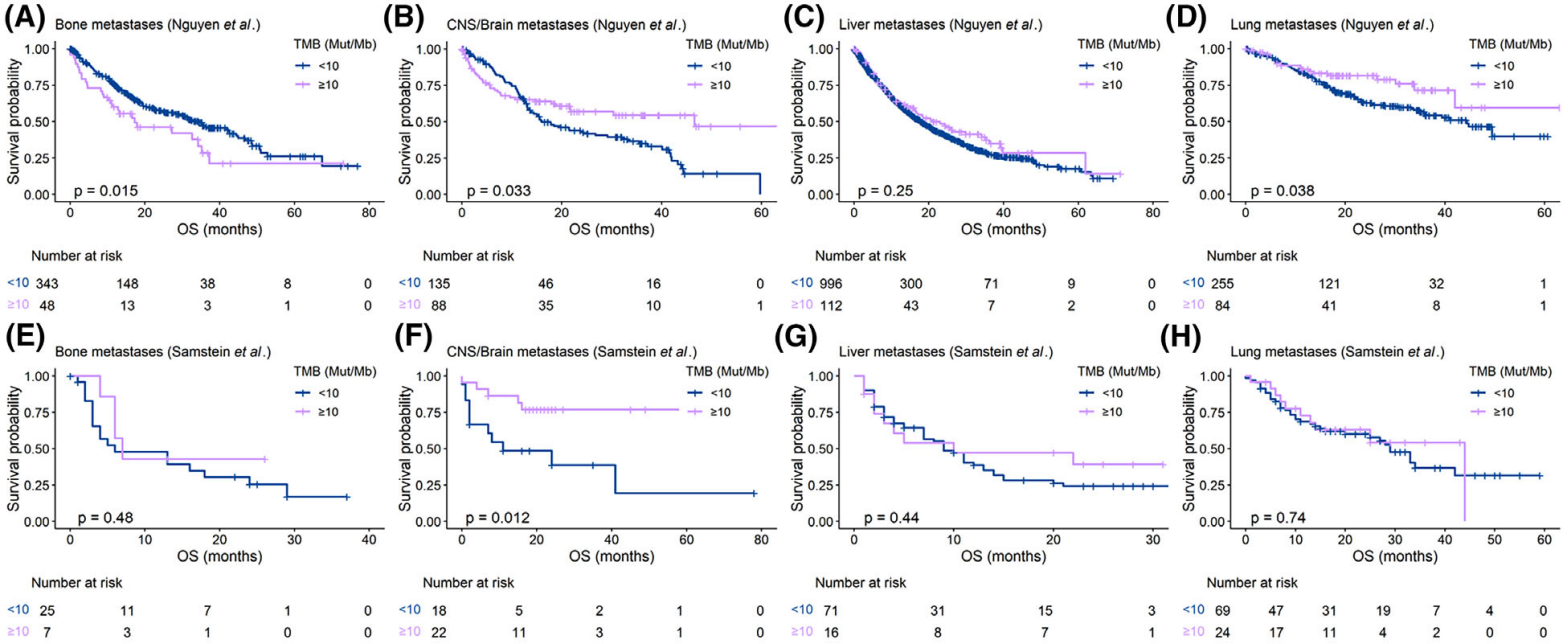
TMB prognostic value across metastatic locations. Kaplan–Meier curves according to TMB measured in bone, CNS/brain, liver, and lung metastases for OS in patients of the Nguyen *et al*. (A–D) and in the Samstein *et al*. (E–H) dataset, respectively. TMB was dichotomized using a cut‐off value of 10 Mut/Mb. *P*‐value was obtained using log‐rank test. OS, Overall survival; *P*, *P*‐value; TMB, tumor mutational burden.

We then investigated whether the observed differences in TMB across sample sites could influence patient survival. To this end, we evaluated the prognostic value of TMB in the analyzed patient cohorts. As expected, higher TMB was associated with longer overall survival (OS), regardless of whether TMB was measured across all samples, only on metastatic lesions, or exclusively in primary tumor samples (Table 1 and Fig. S5A–C). However, when focusing on specific metastatic locations, we observed that higher TMB measured in CNS/brain and lung metastases was associated with improved prognosis, whereas elevated TMB in bone metastases correlated with poorer survival outcomes (Fig. 7A–D).

**Table 1:** Univariate Cox regression survival analysis of TMB for OS in the Nguyen *et al*. dataset and the Samstein *et al.* dataset. Results shown reflect comparison of High vs. Low TMB using a cut‐off value of 10 Mut/Mb, measured on all samples, on primary tissue, on metastatic tissue, or on bone, CNS/brain, liver, and lung metastases for the Nguyen *et al.* and Samstein *et al.* datasets. CI, 95% confidence interval; CNS, central nervous system; HR, Hazard ratio; Mb, megabase; Mut, mutation; OS, overall Survival; *P*, *P*‐value; TMB, tumor mutational burden.

TMB high vs. low	Nguyen *et al*. dataset	Samstein *et al*. dataset
	HR (95% CI, *P*‐value)	HR (95% CI, *P*‐value)
All samples	0.79 (0.71–0.88, *P* < 0.001)	0.68 (0.51–0.90, *P* = 0.006)
Metastatic samples	0.79 (0.66–0.95, *P* = 0.010)	0.56 (0.37–0.85, *P* = 0.006)
Primary tumor samples	0.78 (0.69–0.89, *P* < 0.001)	0.79 (0.54–1.15, *P* = 0.215)
Bone metastasis samples	1.63 (1.10–2.43, *P* = 0.015)	0.68 (0.23–2.03, *P* = 0.484)
CNS/brain metastasis samples	0.65 (0.44–0.97, *P* = 0.035)	0.28 (0.10–0.81, *P* = 0.018)
Liver metastasis samples	0.86 (0.66–1.12, *P* = 0.250)	0.76 (0.37–1.55, *P* = 0.445)
Lung metastasis samples	0.59 (0.35–0.98, *P* = 0.040)	0.89 (0.44–1.79, *P* = 0.738)

In the Samstein *et al*. dataset, including patients treated with immunotherapy, we confirmed the prognostic role of TMB (Table 1 and Fig. S5D–F). We subsequently evaluated whether the prognostic value of TMB varied according to the metastatic tissue analyzed (Fig. 7E–H). Higher TMB was associated with improved OS when considering all metastatic samples, as well as specifically in CNS/brain metastases. However, no significant association between TMB and OS was observed in other metastatic locations.

## 4 Discussion

In this pan‐cancer study, we explored whether genomic mutational profiles influence metastatic dissemination patterns. Specifically, we wondered whether similarities in mutational landscapes correlate with metastatic behavior and contribute to organ‐specific tropism.

To address this question, we applied two complementary approaches. First, based on the assumption that driver gene mutations are shared across tumor samples, we analyzed recurrently mutated genes. Second, to capture the global mutational landscape—including both driver and passenger mutations—we employed TMB as a proxy for global mutational load.

One of the most compelling findings from our TMB analysis was the consistently higher TMB observed in CNS/brain metastases across two independent patient cohorts. This pattern was particularly evident in specific cancer types such as melanoma and NSCLC, in agreement with previous studies [18, 19, 20]. On the one hand, this may reflect a tumor phenotype characterized by greater complexity and plasticity. On the other hand, this may be related to the immune‐privileged nature of the CNS that may enable tumor clones with high TMB and increased neoantigen load to escape immune surveillance. Previous studies have reported reduced T‐cell infiltration in CNS/brain metastases compared to their matched primary tumors, which could facilitate the persistence of highly mutated clones in the brain microenvironment [21, 22, 23]. Our analysis also revealed increased TMB in lung metastases in the Nguyen *et al*. dataset, supporting the hypothesis that these lesions may be more immunogenic than metastases in other organs. This observation is consistent with findings from our previous study [11], suggesting that lung metastases may possess both genomic and transcriptomic features associated with increased immunogenicity. However, this trend was not replicated in the Samstein *et al*. dataset, highlighting inter‐cohort variability. Such discrepancies are echoed in current literature. For instance, Qiu *et al*. found higher TMB in pulmonary metastases compared to CRC primary tumors. However, a pan‐cancer analysis conducted by Papillon‐Cavanagh did not observe significant TMB differences between lung metastases and primary tumors, although a tendency was observed in certain cancer types such as melanoma [19, 24].

We also observed that variations in TMB across metastatic locations were cancer‐type dependent, in line with previous studies [18, 19, 20]. As expected, melanoma, NSCLC, and hypermutated CRC exhibited higher TMB, consistent with the existing literature [25]. Interestingly, these high TMB tumors tend to metastasize more frequently to the CNS/brain, whereas less immunogenic cancers—including prostate, renal, and breast cancers—preferentially metastasize to bone [17, 22]. These patterns raised the hypothesis that the immune landscape of each metastatic site may act as a selective barrier, favoring expansion of tumor clones with specific genomic profiles.

To our knowledge, this is the first study reporting differences in the prognostic value of TMB based on metastatic locations, independently of the primary cancer types. These findings might have important implications for patient stratification and therapeutic decision‐making, particularly in the context of immunotherapy, where genomic biomarkers are increasingly used to guide precision medicine. The genomic heterogeneity observed across metastatic locations may also help to explain the phenomenon of dissociated responses to immunotherapy [26, 27]. In both datasets, higher TMB in CNS/brain metastases was associated with improved prognosis. This observation aligns with previous studies reporting that CNS/brain metastases with lower TMB were associated with poorer outcomes in lung cancer patients [18, 28], providing rationale for immunotherapy in patients with high TMB CNS/brain metastasis. Although most clinical trials initially excluded patients with CNS/brain metastases, accumulating evidence supports the efficacy of immune checkpoint inhibitors in these patients. A similar association was observed in lung metastases, reinforcing the idea that these sites may be more immunogenic and thus more responsive to immunotherapy than other metastatic locations, such as liver, which is characteristically resistant to immune checkpoint blockade [26, 29, 30]. The approval of a TMB cut‐off value of 10 Mut/Mb as a tumor‐agnostic biomarker for pembrolizumab in solid tumors has been controversial, as TMB is influenced by cancer type and various technical and biological factors [14, 31, 32, 33]. Our results propose metastatic location as an additional critical source of variability, echoing findings from prior studies [18, 19, 20]. Notably, the prognostic value of TMB differed depending on whether it was measured in metastatic or primary tumor tissue.

Our findings also suggested that metastatic tumors located in the same organ do not consistently share specific mutations. Instead, the mutational patterns observed in metastatic samples seem to be primarily dictated by the origin of the primary tumor, rather than the secondary‐invaded organ. None of the multivariate models tested could accurately predict metastatic location based solely on mutational profiles. These observations suggested that the adaptation to the metastatic microenvironment may be predominantly governed by transcriptional regulation rather than by genomic mutations—a hypothesis supported by our previous work [11] and consistent with existing literature [34, 35, 36].

However, when stratifying by cancer type, we observed certain site‐specific trends. In CRC and pancreatic cancers, *KRAS* mutations appeared more frequently in lung, CNS/brain and intra‐abdominal metastases; compared to liver metastases or primary tumor samples, underscoring the potential role of the *KRAS* pathway in microenvironmental adaptation [37, 38, 39, 40]. Additionally, lung metastases from melanoma patients consistently harbored a higher number of mutated genes, suggesting increased genomic complexity. Notably, metastatic and non‐metastatic primary tumor samples did not cluster together in NSCLC, pancreatic, ovarian, endometrial, and bladder cancer. This points to mutational evolution over time, with primary tumors from metastatic patients resembling metastatic lesions more closely.

Regarding specific mutations, some of our findings had previously been reported in the literature. For example, *MAP2K4* mutations have been associated with CNS/brain metastasis in breast cancer [41]. In ovarian cancer, *ARID1A* appeared more frequently mutated in primary tumor samples than in metastases, where loss of mutation was common. Intriguingly, ovarian metastases from breast cancer were enriched for *ARID1A* mutations, suggesting its potential role in the ovarian microenvironment. In addition, we observed *ARID1A* mutations more frequently in metastatic breast cancer than in primary non‐metastatic tumors, consistent with its proposed role in promoting metastasis [42]. A recent study by Lusby *et al*. reported a genetic signature of 177 genes as potential drivers of metastasis across different cancer types. Among these, only the *CDH1* gene was found more frequently mutated in ovarian metastases from breast cancer in our dataset. However, it is important to note that this pan‐cancer study did not include metastatic samples or site‐specific comparisons [43]. Giannou *et al*. demonstrated that cancers harboring *NRAS* mutations spontaneously metastasize to the lungs, an event associated with inflammation [44]. This is in line with our previous findings linking inflammation to lung colonization [11]. Consistently, in the Nguyen *et al*. dataset, *NRAS* mutations were enriched in lung metastases from NSCLC, CRC cancer, and melanoma patients, but also in other metastatic sites, suggesting it may not be lung‐specific.

Among the limitations of this study, primary and metastatic samples were not patient‐matched. Furthermore, sample distribution across metastatic location was uneven, probably influenced by metastatic organotropism and differences in accessibility between metastases and primary tumors. Moreover, these datasets included both distant and regional lymph nodes and lacked information regarding cancer subtypes. Our analysis was based on a targeted gene panel rather than whole‐exome sequencing. While this approach may limit the comprehensiveness of mutation detection, targeted gene panels are more feasible for clinical implementation and widely used in routine practice. The predictive accuracy of our models might be further improved by evaluating specific mutations rather than mutated genes. Additionally, the differences observed between datasets are noteworthy and deserve further investigation. For instance, the cohort from Samstein *et al*. is inherently biased, as patients were selected based on eligibility for immunotherapy. Furthermore, differences in sample size and representation across datasets may have influenced the results and should be considered when interpreting the findings.

## 5 Conclusions

In conclusion, our findings suggest that TMB may play a more significant role in metastatic organotropism in addition to driver mutational background. No robust mutational patterns were identified across different metastatic locations and cancer types. In contrast, TMB levels and their prognostic impact varied depending on the metastatic location, particularly in CNS/brain metastases. These findings may be pivotal in refining immunotherapy strategies and guiding clinical decision‐making in the era of precision oncology.

## Conflict of interest

The authors declare no conflict of interest.

## Author contributions

CS and RSP conceived and designed the project. EC, AMM, and RSP collected and curated the data. EC, AMM, MSP, and RSP analyzed and interpreted the data. All authors have written the manuscript. All authors have been involved in the final approval of the manuscript.

## Supporting information


**Figure S1.** Barplots showing frequency of patients with mutations in the top 50 mutated genes for each cancer type (A–I).
**Figure S2.** Frequency of metastatic locations by cancer type (A–I) in the Nguyen *et al*. dataset.
**Figure S3.** TMB patterns across metastatic locations by cancer types.
**Figure S4.** Frequency of patients with high and low TMB across sample sites by cancer types.
**Figure S5.** Kaplan‐Meier curves according to TMB measured in all samples, primary tissue samples, or metastasis tissue samples in (A–C) Nguyen *et al*. and (D–F) Samstein *et al*. dataset.

**Table S1.** Baseline characteristics of patients from the Nguyen *et al*. dataset.

**Table S2.** Baseline characteristics of patients from the Samstein *et al*. dataset.

**Table S3.** Frequency of metastatic locations by cancer type in the Nguyen *et al*. dataset.

**Table S4.** Performance metrics of all the constructed models.

**Table S5.** Frequency of mutations across sample sites and cancer types for the top 20 genes. *N, number*.

**Table S6.** TMB across sample sites in the Nguyen *et al*. dataset and the Samstein *et al*. dataset.

**Table S7.** TMB across sample sites and cancer types in the Nguyen *et al*. dataset and the Samstein *et al*. dataset.

## Data Availability

The data that support the findings of this study are available in cBioPortal at https://www.cbioportal.org/study/summary?id=tmb_mskcc_2018 (id = tmb_mskcc_2018) and https://www.cbioportal.org/study/summary?id=msk_met_2021 (id = msk_met_2021). These data were derived from the following scientific articles: doi: 10.1038/s41588‐018‐0312‐8 and doi: 10.1016/j.cell.2022.01.003.
