# Supplementary material for: Tumor mutational burden as a determinant of metastatic dissemination patterns
Source: Mol Oncol. 2026 Jan 27;20(5):1364–78. doi: 10.1002/1878-0261.70200 (PMC13155152; doi:10.1002/1878-0261.70200)
Supplement: Supplementary file 1 — Figure S1. Barplots showing frequency of patients with mutations in the top 50 mutated genes for each cancer type (A–I). Figure S2. Frequency of metastatic locations by cancer type (A–I) in the Nguyen et al. dataset. Figure S3. TMB patterns across metastatic locations by cancer types. Figure S4. Frequency of patients with high and low TMB across sample sites by cancer types. Figure S5. Kaplan‐Meier curves according to TMB measured in all samples, primary tissue samples, or metastasis tissue samples in (A–C) Nguyen et al. and (D–F) Samstein et al. dataset. [file MOL2-20-1364-s004.zip › RSanz_SupportingInformation_Legends.docx]

**SUPPORTING INFORMATION**

**Supplementary tables**

**Supplementary table 1**. Baseline characteristics of patients from the Nguyen *et al*. dataset.

**Supplementary table 2**. Baseline characteristics of patients from the Samstein *et al*. dataset.

**Supplementary table 3**. Frequency of metastatic locations by cancer type in the Nguyen *et al.* dataset.

**Supplementary Table 4**. Performance metrics of all the constructed models.

**Supplementary Table 5.** Frequency of mutations across sample sites and cancer types for the top 20 genes. *N, number.*

**Supplementary Table 6**. TMB across sample sites in the Nguyen *et al*. dataset and the Samstein *et al.* dataset.

**Supplementary Table 7.** TMB across sample sites and cancer types in the Nguyen *et al*. dataset and the Samstein *et al.* dataset.

**Supplementary figures**

**Supplementary Figure 1**. **Barplots showing frequency of patients with mutations in the top 50 mutated genes for each cancer type (A-I).** Vertical dashed lines mark the top20 genes, which have been selected as threshold for PCA and heatmap representations. Sample size was 18913 patients.

**Supplementary Figure 2**. **Frequency of metastatic locations by cancer type (A-I) in the Nguyen *et al.* dataset**. Pie charts representing the frequency of samples from each metastatic location for A) bladder, B) breast, C) colorectal, D) endometrial, E) melanoma, F) Non-Small Cell Lung, G) ovarian, H) pancreatic, and I) prostate cancer. Category others includes metastases in the biliary tract, bladder/urinary tract, bowel, breast, female genital tract (excluding ovary), head and neck, and male genital tract*.* Sample size was 6883 patients. *NSCLC, Non-Small Cell Lung Cancer; CNS, Central Nervous System.*

**Supplementary Figure 3**. **TMB patterns across metastatic locations by cancer types**. Boxplots of log10 TMB (Mut/Mb) according to cancer type and sample site of patients of the Nguyen *et al*. dataset (A) and patients of the Samstein *et al*. dataset (B). Sample size was 8438 (A) and 590 (B) patients. P-value was obtained using Kruskal-Wallis test. *TMB, Tumor Mutational Burden; Mut, Mutation; Mb, Megabase.*

**Supplementary Figure 4**. **Frequency of patients with high and low TMB across sample sites by cancer types.** Barplots showing frequency of high and low TMB according to cancer type and sample site of patients of the A) Nguyen *et al*. and B) Samstein *et al*. dataset. TMB was dichotomized using a cut-off value of 10 Mut/Mb. Sample size was 8438 (A) and 590 (B) patients. P-value was obtained using Fisher’s exact test. *TMB, Tumor Mutational Burden; Mut, Mutation; Mb, Megabase.*

**Supplementary Figure 5**. **Kaplan-Meier curves according to TMB measured in all samples, primary tissue samples, or metastasis tissue samples in A-C) Nguyen *et al*. and D-F) Samstein *et al.* dataset. TMB was dichotomized using a cut-off value of 10 Mut/Mb.** P-value was obtained using log-rank test. *OS, Overall Survival; TMB, Tumor Mutational Burden; p, p-value.*
