# Supplementary figures and images for: Tumor mutational burden as a determinant of metastatic dissemination patterns
Source: Mol Oncol. 2026 Jan 27;20(5):1364–78. doi: 10.1002/1878-0261.70200 (PMC13155152; doi:10.1002/1878-0261.70200)

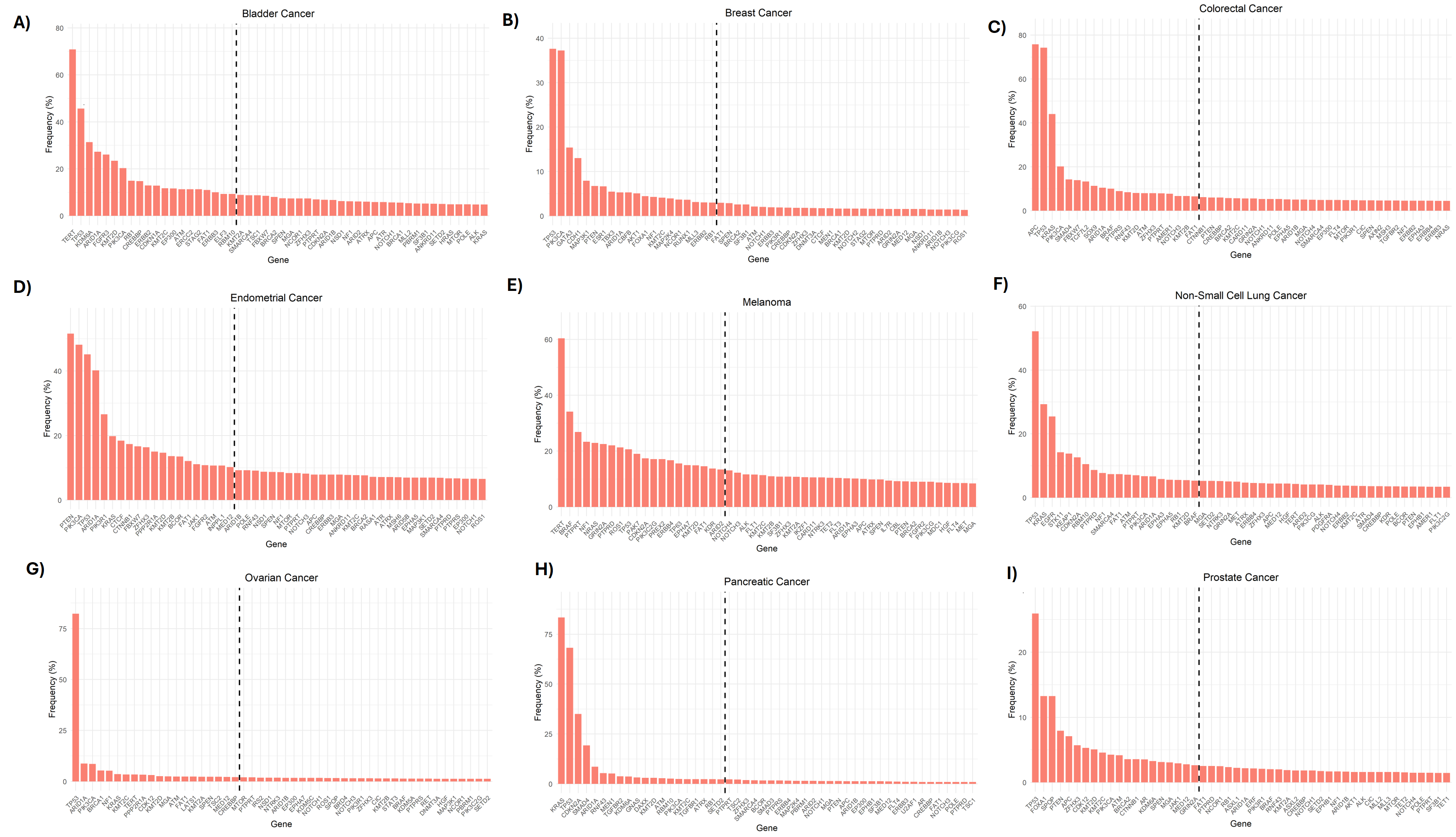

Supplement: Supplementary file 1 — Figure S1. Barplots showing frequency of patients with mutations in the top 50 mutated genes for each cancer type (A–I). Figure S2. Frequency of metastatic locations by cancer type (A–I) in the Nguyen et al. dataset. Figure S3. TMB patterns across metastatic locations by cancer types. Figure S4. Frequency of patients with high and low TMB across sample sites by cancer types. Figure S5. Kaplan‐Meier curves according to TMB measured in all samples, primary tissue samples, or metastasis tissue samples in (A–C) Nguyen et al. and (D–F) Samstein et al. dataset. [file MOL2-20-1364-s004.zip › mol270200-sup-0001-SupplementaryFigure1.tif]

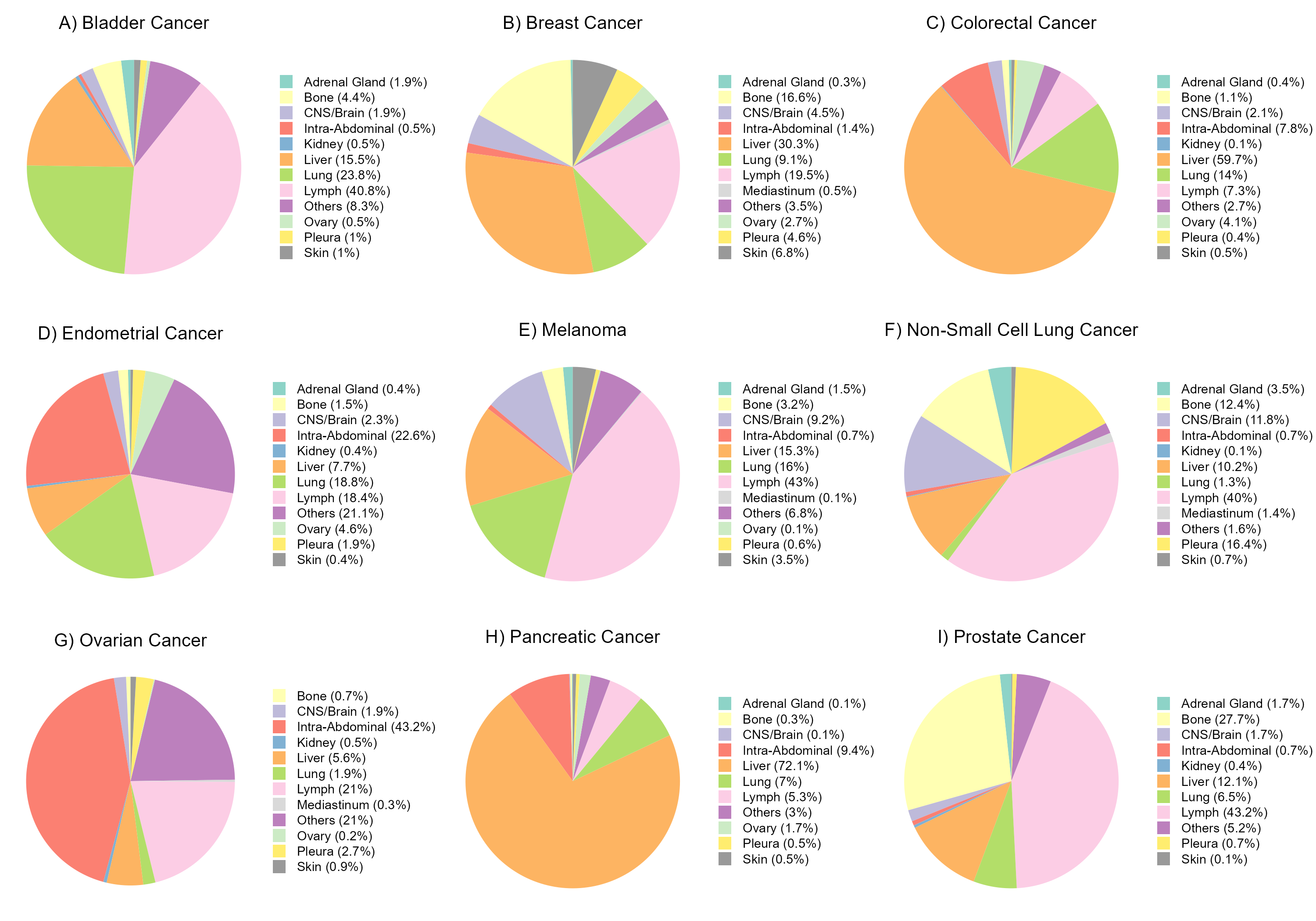

Supplement: Supplementary file 1 — Figure S1. Barplots showing frequency of patients with mutations in the top 50 mutated genes for each cancer type (A–I). Figure S2. Frequency of metastatic locations by cancer type (A–I) in the Nguyen et al. dataset. Figure S3. TMB patterns across metastatic locations by cancer types. Figure S4. Frequency of patients with high and low TMB across sample sites by cancer types. Figure S5. Kaplan‐Meier curves according to TMB measured in all samples, primary tissue samples, or metastasis tissue samples in (A–C) Nguyen et al. and (D–F) Samstein et al. dataset. [file MOL2-20-1364-s004.zip › mol270200-sup-0002-SupplementaryFigure2.tiff]

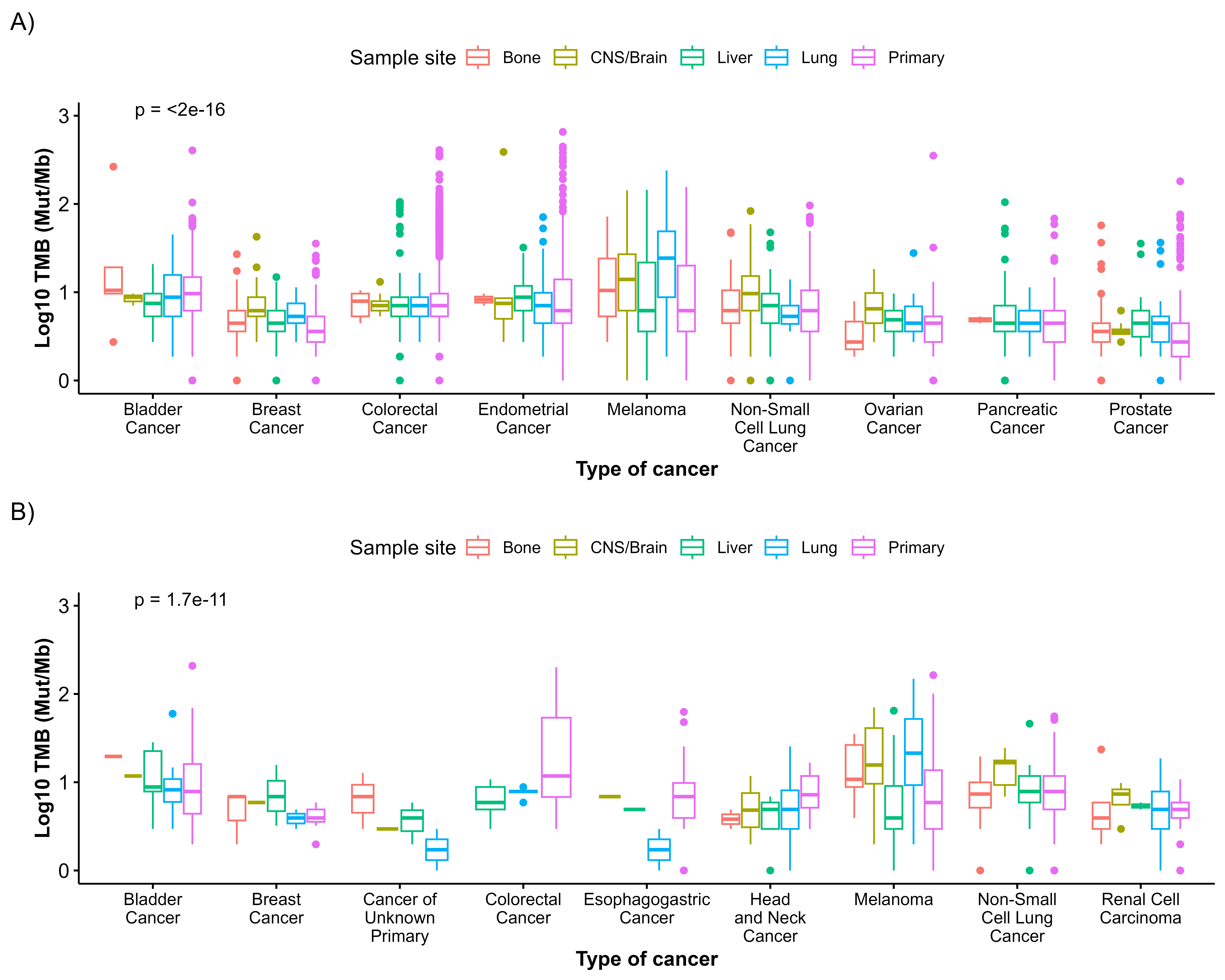

Supplement: Supplementary file 1 — Figure S1. Barplots showing frequency of patients with mutations in the top 50 mutated genes for each cancer type (A–I). Figure S2. Frequency of metastatic locations by cancer type (A–I) in the Nguyen et al. dataset. Figure S3. TMB patterns across metastatic locations by cancer types. Figure S4. Frequency of patients with high and low TMB across sample sites by cancer types. Figure S5. Kaplan‐Meier curves according to TMB measured in all samples, primary tissue samples, or metastasis tissue samples in (A–C) Nguyen et al. and (D–F) Samstein et al. dataset. [file MOL2-20-1364-s004.zip › mol270200-sup-0003-SupplementaryFigure3.tiff]

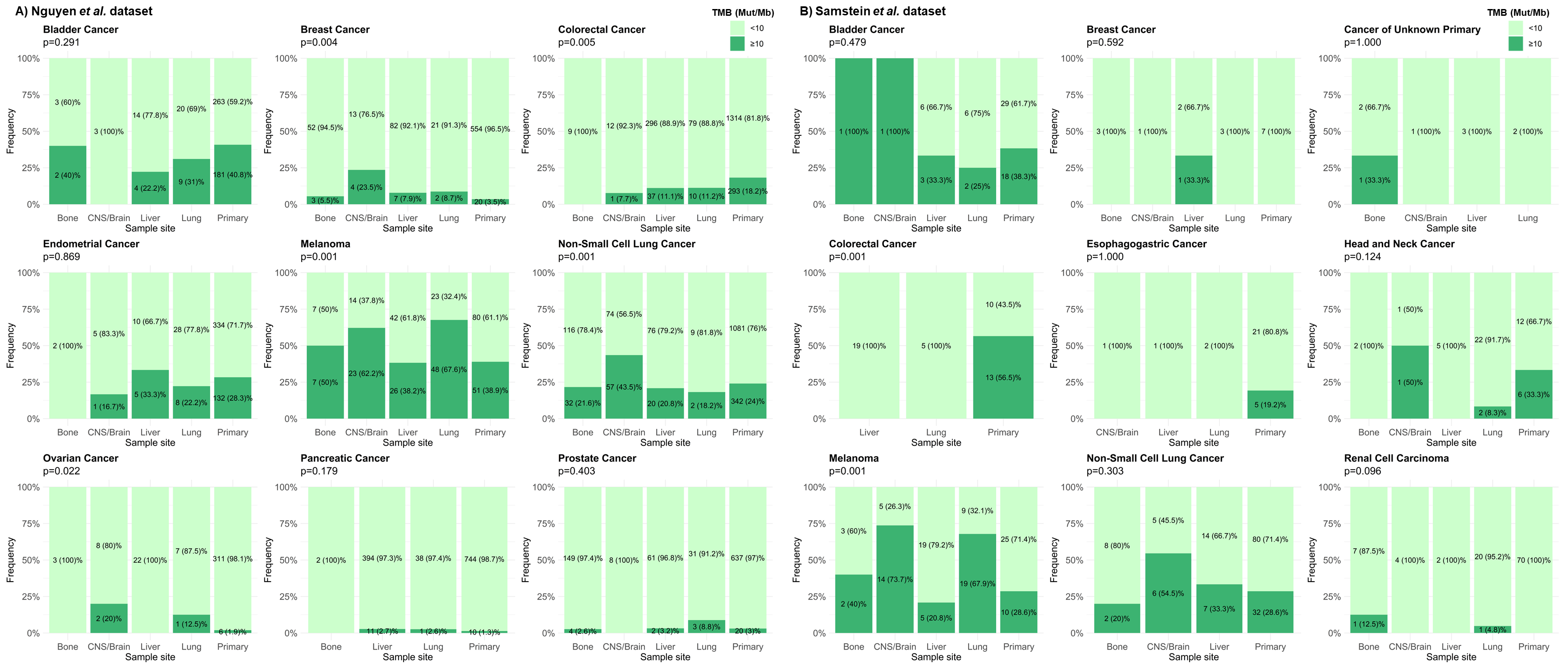

Supplement: Supplementary file 1 — Figure S1. Barplots showing frequency of patients with mutations in the top 50 mutated genes for each cancer type (A–I). Figure S2. Frequency of metastatic locations by cancer type (A–I) in the Nguyen et al. dataset. Figure S3. TMB patterns across metastatic locations by cancer types. Figure S4. Frequency of patients with high and low TMB across sample sites by cancer types. Figure S5. Kaplan‐Meier curves according to TMB measured in all samples, primary tissue samples, or metastasis tissue samples in (A–C) Nguyen et al. and (D–F) Samstein et al. dataset. [file MOL2-20-1364-s004.zip › mol270200-sup-0004-SupplementaryFigure4.tiff]

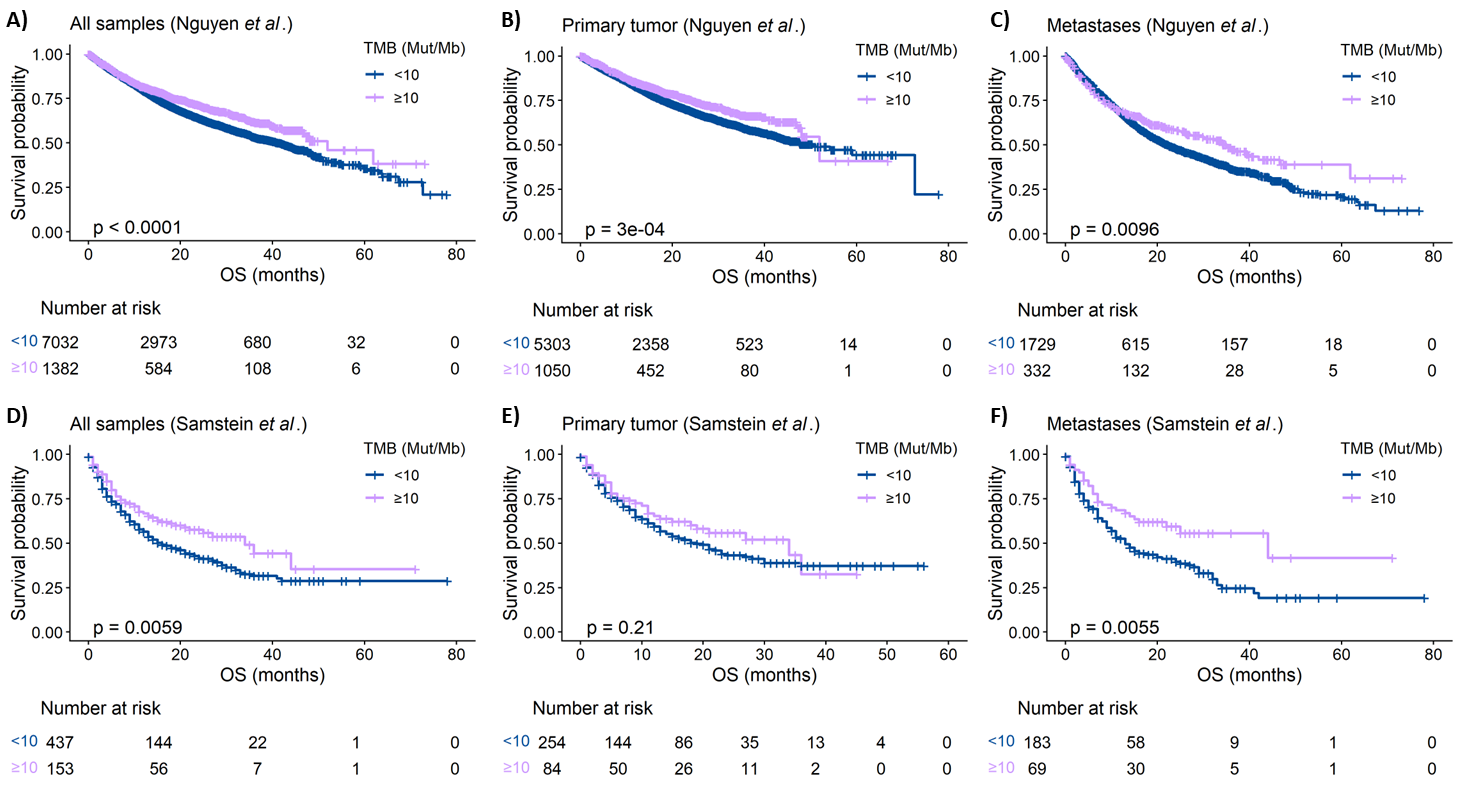

Supplement: Supplementary file 1 — Figure S1. Barplots showing frequency of patients with mutations in the top 50 mutated genes for each cancer type (A–I). Figure S2. Frequency of metastatic locations by cancer type (A–I) in the Nguyen et al. dataset. Figure S3. TMB patterns across metastatic locations by cancer types. Figure S4. Frequency of patients with high and low TMB across sample sites by cancer types. Figure S5. Kaplan‐Meier curves according to TMB measured in all samples, primary tissue samples, or metastasis tissue samples in (A–C) Nguyen et al. and (D–F) Samstein et al. dataset. [file MOL2-20-1364-s004.zip › mol270200-sup-0005-SupplementaryFigure5.tiff]
